# Effectiveness and safety of 30 mg versus 40 mg stavudine regimens: a cohort study among HIV-infected adults initiating HAART in South Africa

**DOI:** 10.1186/1758-2652-15-13

**Published:** 2012-03-12

**Authors:** Mhairi Maskew, Daniel Westreich, Matthew P Fox, Thapelo Maotoe, Ian M Sanne

**Affiliations:** 1 Clinical HIV Research Unit, Department of Medicine, Faculty of Health Sciences, University of the Witwatersrand, Johannesburg, South Africa; 2 Health Economics and Epidemiology Research Office, Department of Medicine, Faculty of Health Sciences, University of the Witwatersrand, Johannesburg, South Africa; 3 Department of Obstetrics and Gynecology and Global Health Institute, Duke University, Durham, NC, USA; 4 Center for Global Health and Development, Boston, USA; 5 Department of Epidemiology, Boston University School of Public Health, Boston, USA; 6 Right to Care, Johannesburg, South Africa; 7 Health Economics and Epidemiology Research Office, Suite 176, Private Bag x2600, Houghton 2041, South Africa

**Keywords:** Antiretroviral toxicity, Stavudine, Resource-limited setting, Cohort study, Virologic failure

## Abstract

**Background:**

As stavudine remains an important and widely prescribed drug in resource-limited settings, the effect of a reduced dose of stavudine (from 40 mg to 30 mg) on outcomes of highly active antiretroviral therapy (HAART) remains an important public health question.

**Methods:**

We analyzed prospectively collected data from the Themba Lethu Clinic in Johannesburg, South Africa. We assessed the relationship between stavudine dose and six- and/or 12-month outcomes of stavudine substitution, failure to suppress viral load to below 400 copies/ml, development of peripheral neuropathy, lipoatrophy and hyperlactatemia/lactic acidosis. Since individuals with a baseline weight of less than 60 kg were expected to have received the same dose of stavudine throughout the study period, analysis was restricted to individuals who weighed 60 kg or more at baseline. Data were analyzed using logistic regression.

**Results:**

Between 1 April 2004 and 30 September 2009, 3910 patients were initiated on antiretroviral therapy (ART) with a recorded stavudine dose and were included in the analysis. Of these, 2445 (62.5%) received a 40 mg stavudine dose while 1565 (37.5%) received 30 mg. In multivariate analysis, patients receiving a 40 mg dose were more likely to discontinue stavudine use (adjusted odds ratio, OR 1.71; 95% confidence limits, CI 1.13-2.57) than those receiving 30 mg by 12 months on ART. Additionally, patients receiving 40 mg doses of stavudine were more likely to report peripheral neuropathy (OR 3.12; 95% CI 1.86-5.25), lipoatrophy (OR 11.8; 95% CI 3.2-43.8) and hyperlactatemia/lactic acidosis (OR 8.37; 95% CI 3.83-18.29) in the same time period. Failure to suppress HIV viral load within 12 months of HAART initiation was somewhat more common among those given 40 mg doses (OR 1.62; 95% CI 0.88, 2.97) although this result lacked precision. Sensitivity analyses accounting for death and loss to follow up generally supported these estimates.

**Conclusions:**

Lower stavudine dosage is associated with fewer reports of several stavudine-associated adverse events and also a lower risk of stavudine discontinuation within the first year on ART.

## Background

Stavudine (d4T) is a nucleoside analogue that has been widely used as part of highly active antiretroviral therapy (HAART). Despite being highly effective [1,2], use of stavudine-based regimens has been eliminated from resource-rich environments due to the poor side effects profile and high rates of adverse events. In particular, stavudine-containing regimens have been associated with metabolic complications, such as dyslipidemias, lipoatrophy and other mitochondrial toxicities, notably peripheral neuropathy and lactic acidosis [3-14]. This led the World Health Organization (WHO) to recommend a lower maximum dose of stavudine for all adults in 2007 [15], and in 2009, to recommend that it no longer be used for initial treatment of HIV infection due to serious side effects [16].

Other antiretroviral agents have been developed and exhibit lower rates of toxicity even among stavudine-experienced individuals. These include improvement of dyslipidaemia on tenofovir [17-19] and lipoatrophy on abacavir [20]. A large placebo-controlled trial comparing stavudine and tenofovir demonstrated reduced rates of investigator-reported lipoatrophy among the tenofovir arm, but no difference in mortality, grade 3 or 4 clinical adverse events, rates of HIV RNA suppression or CD4 count increases between the treatment arms [21]. However, despite changes in country-level antiretroviral guidelines recommending the use of these newer agents, cost has precluded their widespread use in resource-limited settings [6,22]; therefore, more than half of individuals receiving antiretroviral therapy have stavudine as part of their first-line regimens in low- and middle-income countries [23]. Alternate strategies need to be sought to reduce stavudine-associated morbidity in these settings.

Stavudine has traditionally been prescribed in weight-dependent doses: 30 mg for those weighing less than 60 kg, and 40 mg for those at or above 60 kg. However, using 30 mg of stavudine may reduce adverse events and toxicities without reducing efficacy regardless of body weight. In addition to the studies noted, small-scale randomized trials have suggested a trend towards a decrease in plasma lipids and improvements in peripheral wasting with lower-dose stavudine [17,24]. However, in order to be a useful strategy, the optimal dose of stavudine that limits drug-induced toxicity while maintaining virologic potency needs to be investigated in large populations in resource-limited settings where the clinical and economic pressure to continue stavudine use is high.

We evaluated the effectiveness and tolerance of a dose of 30 mg versus 40 mg stavudine as part of HAART regimens in an HIV-infected population receiving HAART.

## Methods

### Study site and data collection

Themba Lethu Clinic is an urban antiretroviral rollout site based at Helen Joseph Hospital, a public hospital in Johannesburg, South Africa. Since the national rollout of antiretroviral drugs in 2004, the clinic has enrolled more than 23,000 HIV-infected adults and currently provides free antiretroviral therapy to more than 16,000 of these patients according to the South African national Department of Health's guidelines on rollout of antiretroviral therapy [25]. Standard first-line therapy in South Africa at the time of this analysis consisted of stavudine, lamivudine and efavirenz (or nevirapine). Treatment was initiated at CD4 counts of 200 cells/mm^3 ^or less or a clinical WHO stage III/IV [26].

Clinical, demographic and laboratory data is collected prospectively. Patient information is recorded electronically by attending nurses and clinicians during clinical visits, while a team of trained data capturers conduct quality assurance checks. Prior to initiating HAART, all patients receive adherence counselling and have a staging baseline CD4 count taken. Patients return monthly to pick up medication and are scheduled for clinical assessment by a doctor at one, two and four months after initiation of HAART or as clinically indicated. After four months, patients return for clinical visits every six months or as clinically indicated. Initial assessment of treatment success (as determined by CD4 count response and suppression of HIV viral load) is measured at the four-month visit and six monthly thereafter.

We analyzed previously collected data for all HAART-naïve adult patients initiating stavudine-containing HAART between 1 April 2004 and 30 September 2009, and followed up until 1 April 2010, allowing for a minimum of six months of follow up for each subject. Women who were pregnant at initiation were excluded as they are initiated on HAART at higher CD4 counts, have different HAART regimens [25] and have variable CD4 counts (due to the hemodilution effect of pregnancy) compared with the general population. With few exceptions, individuals weighing less than 60 kg at HAART initiation received a 30 mg dose of stavudine throughout follow up. Thus, we studied only individuals with baseline weights of 60 kg or more, as these were the only subjects who saw a variation of dose in the study period (see the next section, Exposure).

### Exposure

The exposure was defined as the dose of stavudine prescribed at baseline. Until the end of 2007, stavudine dosing was weight-based in South Africa, with Department of Health guidelines prescribing 30 mg doses for those weighing less than 60 kg and 40 mg doses for those weighing 60 kg or more. From October 2007, a universal 30 mg dose was introduced and 40 mg tablets of stavudine were withdrawn from the clinic. Thus, we limited the study to those with baseline weights of 60 kg or more, and the dose received was defined by calendar date (40 mg in the pre-change period, 30 mg in the post-change period).

Dosing was essentially concordant with protocol: a validation review of 50 records of randomly selected subjects with baseline weights of 60 kg or more before January 2008 yielded a high concordance (> 95%) of recorded dose with protocol. To ensure precise definitions, we instituted a "wash-out" period from July 2007 until March 2008. Individuals initiated onto treatment during this period were excluded from the study. We conducted an intent-to-treat analysis by assuming that dose did not change in the first 12 months of follow up.

### Outcomes

We considered five outcomes at up to two time points. These outcomes were: 1) virologic failure; 2) discontinuation of stavudine; 3) peripheral neuropathy evaluated at both six and 12 months; 4) lipoatrophy and 5) hyperlactatemia/lactic acidosis, (the last two were evaluated at 12 months only due to very few cases occurring in the first six months). Virologic failure was defined as failure to suppress virus to < 400 copies/ml by six or 12 months; individuals without a viral load measurement in the first six or 12 months were excluded from analysis of this last outcome. A patient was considered to have experienced peripheral neuropathy, lipoatrophy or hyperlactatemia/lactic acidosis if, during the study period, a clinical diagnosis was recorded on the patient record by the attending physician. The procedures for detecting and managing hyperlactatemia and lactic acidosis remained constant throughout the study period. This included clinical screening and formal laboratory testing for lactate levels. All individuals with raised lactate levels were referred to the Helen Joseph Hospital for further investigation and management, including arterial blood gas status, where appropriate.

### Statistical methods

Characteristics of patients by dose of stavudine were described using simple descriptive statistics. Effects of the exposure on the outcomes (as we have listed) were calculated using logistic regression to estimate odds ratios. Since the incidence of most outcomes were low (≤ 10%), those odds ratios generally approximated risk ratios. All analyses controlled for confounding by baseline weight, height, age, gender, ethnicity, employment status, haemoglobin, CD4 count, WHO stage, tuberculosis status, reported baseline peripheral neuropathy, calendar date and whether clinical care was free at baseline. We controlled for both age and weight using relatively unrestrictive four-knot restricted cubic splines.

We performed two sensitivity analyses to account for potential selection bias by death or loss to follow up in the six-month analyses. We first implemented inverse probability of censoring weights [27] and, second, included all death and loss-to-follow-up events as evidence of a poor outcome (e.g., stavudine substitution, incident peripheral neuropathy, or virologic failure).

Use of Themba Lethu Clinic data was approved by the Human Research Ethics Committee of the University of the Witwatersrand. Approval for analysis of de-identified data was also granted by the Boston University and Duke University Institutional Review Boards.

## Results

There were 3910 patients who met the inclusion criteria. Of these, 63.5% (n = 2445) initiated HAART prior to 1 July 2007 and received 40 mg stavudine, while the remaining 37% (n = 1465) initiated HAART after 1 March 2008 and thus had 30 mg stavudine doses (Table 1). Of these patients, 42% (n = 1645) were male; median (interquartile range, IQR) baseline weight was 67 kg (63-75); and median (IQR) age was 37 years (32-43). Seven percent (n = 256) of patients either died (n = 137) or were lost to follow up (n = 119) within the first six months on HAART.

**Table 1:** Characteristics of 3910 individuals initiating HAART at Themba Lethu Clinical by baseline dose of stavudine

Baseline characteristic	Dose of stavudine	
	
	40 mg *n = 2445*	30 mg *n = 1465*
Weight†	67.0 (62.8-74.0)	68.1 (63.6-75.6)
Age†	37 (32-43)	38 (33-44)
Male gender	1017(41.6)	628 (42.9)
African ethnicity	2,348 (96.0)	1,416 (96.7)
Employed	1,130 (46.2)	855 (58.4)
Tuberculosis	364 (14.9)	155 (10.6)
Haemoglobin (low)	823 (35.0)	433 (29.8)
WHO stage III or IV	956 (39.1)	356 (24.3)
CD4 count (cells/mm^3^)†	97 (39-163)	137 (66-192)
Efavirenz-based HAART	2203 (90.1)	1296 (88.5)
Nevirapine-based HAART	193 (7.9)	116 (7.9)

All figures expressed as count (%) unless noted. † Expressed as median (interquartile range)

Only 71% (n = 2785) of patients had a viral load taken within six months and 80% (n = 3139) within 12 months of initiating HAART. At six and 12 months, missing data was similar between the 30 mg and 40 mg dosing groups: 29.2% vs. 28.5% at six months, and 19.9% vs. 21.1% at 12 months. At six months, 1037 had a viral load measurement in the 30 mg group, and 1748 in the 40 mg group. Of these, a total of 177 (6%) failed to suppress virus: 44 (4.2%) in the 30 mg group and 133 (7.6%) in the 40 mg group by 12 months (Table 2). At 12 months, 1171 had a viral load measurement in the 30 mg group, and 1968 in the 40 mg group. Of these, a total of 266 (8.5%) failed to suppress virus: 58 (5.0%) in the 30 mg group and 208 (10.6%) in the 40 mg group by 12 months (Table 2). Failure to suppress within six months of HAART initiation was more common among those given 40 mg than those given 30 mg in crude analyses (OR = 1.86; 95% CI 1.31-2.64). In adjusted analyses, failure to suppress was somewhat more common among those given 40 mg than those given 30 mg at six (OR 1.69; 95% CI 0.81-3.52) and 12 months (OR 1.62; 95% CI 0.88-2.97), although neither result was statistically significant. Neither CD4 count nor other indicators of disease status were independently predictive of virologic failure at either six or 12 months.

**Table 2:** Crude and adjusted odds ratios^† ^comparing 40 mg (n = 2445) and 30 mg (n = 1465) dose of stavudine on outcomes

Outcome Exposure		N (%)	**Crude OR**^†^ (95% CI)	**Adjusted OR **^††^ (95% CI)
**Six months**				
Virologic failure	30 mg	44 (4.2%)	1	1
	40 mg	133 (7.6%)	1.86 (1.31-2.64)	1.69 (0.81-3.52)
Stavudine discontinuation	30 mg	60 (4.1%)	1	1
	40 mg	135 (5.5%)	1.37 (1.00-1.87)	0.87 (0.45-1.71)
Peripheral neuropathy	30 mg	31 (2.1%)	1	1
	40 mg	220 (9.0%)	4.57 (3.12-6.70)	3.17 (1.71-5.90)
**Twelve months**				
Virologic failure	30 mg	58 (5.0%)	1	1
	40 mg	208 (10.6%)	2.27 (1.68-3.06)	1.62 (0.88-2.97)
Stavudine discontinuation	30 mg	158 (10.8%)	1	1
	40 mg	413 (16.9%)	1.68 (1.38-2.05)	1.71 (1.13-2.57)
Peripheral neuropathy	30 mg	41 (2.8%)	1	1
	40 mg	357 (14.6%)	5.94 (4.27-8.26)	3.12 (1.86-5.25)
Lipoatrophy	30 mg	4 (0.3%)	1	1
	40 mg	81 (3.3%)	12.5 (4.6-34.2)	11.8 (3.2-43.8)
Hyperlactemia/lactic acidosis	30 mg	15 (1.0%)	1	1
	40 mg	179 (7.3%)	7.64 (4.49-12.99)	8.37 (3.83-18.29)

^†^OR = Odds ratios estimated using logistic regression models

^††^Estimates adjusted for baseline weight, height, age, gender, ethnicity, employment status, haemoglobin, CD4 count, WHO stage, tuberculosis status, reported baseline peripheral neuropathy, calendar date, and whether clinical care was free at baseline

About 8% (n = 320) of patients experienced a switch in HAART regimen within six months of HAART initiation, and a total of 19% (n = 752) did so by 12 months (Table 2). In both cases, a clear majority of these involved a switch to a non-stavudine-containing HAART regimen (n = 195 and n = 571, respectively). Looking at the 12-month stavudine substitution outcome, of the 571 non-stavudine-containing regimens, 16% (n = 92) contained TDF; a substantially greater percentage of those were among people receiving a 30 mg dose (39%, compared with 8% in the 40 mg dose group).

Crude analyses are shown in Table 2; in adjusted analysis, patients started on a 40 mg dose were no more likely to discontinue stavudine within six months compared with those started on a 30 mg dose (OR 0.87; 95% CI 0.45-1.71), but at 12 months, a 40 mg dose was associated with increased risk (OR 1.71; 95% CI1.13-2.57). Limiting the analysis to only those who received a non-TDF-based regimen gave an adjusted odds ratio of 2.22 (95% CI 1.42-3.48). Among the 571 initial drug regimens in which stavudine was substituted within 12 months, the leading cause for substitution was peripheral neuropathy (n = 156), and a total of 63% were for specifically toxicity-related reasons. Toxicity was more common as a listed reason for stavudine substitution among those receiving a 40 mg dose (76%) than among those receiving a 30 mg dose (30%).

About 6% (n = 251) of patients reported peripheral neuropathy within six months of HAART initiation and 10% (n = 398) by 12 months (Table 2). At both time periods and after adjustment for confounding, the risk for peripheral neuropathy was substantially elevated among those who received a 40 mg dose of stavudine at baseline compared with those receiving 30 mg; the adjusted odds ratios were similar at six and 12 months (3.17 and 3.12, respectively; see Table 2).

Lipoatrophy was reported in less than 2% (n = 81) of subjects by 12 months, and only four of those subjects had initiated HAART on a 30 mg dose (Table 2). The adjusted odds ratio comparing a 40 mg dose with a 30 mg dose for this outcome was 11.8 (95% CI 3.2-43.8). The combined outcome of hyperlactatemia and lactic acidosis was reported in 5% (n = 194) of subjects, and only 15 of those subjects had initiated HAART on a 30 mg dose (Table 2). The adjusted odds ratio for this combined lactate outcome was 8.37 (95% CI 3.83-18.29). In the 30 mg group, all of these events were hyperlactatemia. In the 40 mg group, there were 60% (108 cases) hyperlactatemia, and 40% (71 cases) lactic acidosis. Under a Poisson distribution, the upper 95% confidence limit for incidence of lactic acidosis among subjects with 40 mg dose is approximately 3/1465 or 0.002.

In sensitivity analysis of the six-month outcomes, implementation of inverse probability of censoring weights had almost no effect on point estimates (a less than 0.05 change in odds ratio in all cases). Counting censoring (that is, dead or lost to follow up) as a negative outcome resulted in a qualitatively different result only for the effect of dose on peripheral neuropathy; the odds ratio for the effect of a 40 mg dose on incident peripheral neuropathy in this analysis was 2.15 (95% CI 1.38-3.35). Additionally, we note that limiting the six-month adjusted analyses to those who were least likely (due to timing) to have switched stavudine doses within those first six months of treatment slightly increased the effect of dose on risk of failure to 1.97 (95% CI 0.91-4.25) and did not meaningfully affect other six-month outcomes.

We also noted that patients not receiving EFV or NVP were largely on Kaletra (lopinavir-ritonavir). In further sensitivity analysis, the exclusion of these individuals reduced precision slightly, but did not meaningfully affect point estimates. The estimate for virologic failure at 12 months moved from 1.62 (95% CI 0.88-2.97) to 1.64 (0.89-3.03) when limited to only those with NPV- or EFV-based regimens. Similarly, the estimate for stavudine discontinuation went from 1.71 (1.13-2.57) to 1.73 (1.14-2.61) and for peripheral neuropathy from 3.12 (1.86-5.25) to 3.14 (1.86-5.30).

## Discussion

Despite a well-described toxicity profile and several alternate drug options, stavudine remains widely prescribed in developing countries [28,29]. Recent estimates suggest that 60% of individuals receiving antiretroviral therapy have stavudine as part of their first-line regimens in low- and middle- income countries [23]. Stavudine use has largely ceased in resource-rich settings and WHO guidelines no longer recommend its use [16]. Probably due to this, little attention is currently being paid to questions that are relevant to countries still using this drug, such as determining the optimal dose and the effect of a reduced dose on HAART outcomes [29]. In resource-limited settings, the cost and virologic potency of stavudine, coupled with the limited need for intensive laboratory-based monitoring has meant that stavudine remains an important drug in the rapid scale up of antiretroviral therapy in countries with the greatest need.

Over the past few years, several stavudine-dosing schemes have been used: a universal 40 mg dose [30]; weight-based dosing [26,31]; and a universal 30 mg dose [16]. Each of these dosing schemes has potential advantages and disadvantages: higher doses may be more effective at controlling viral load, but have potentially poorer adherence, while lower doses may reduce side effects, but be less effective at suppressing virus. In this study, we sought to address the relative advantages and disadvantages of stavudine dose.

Stavudine use has been shown to result in frequent (up to 30%) discontinuation of drug use due to multiple adverse events, including peripheral neuropathy, dyslipidemias, lipoatrophy and mitochondrial toxicity [1,3-9,14,22,30-34]. There are, however, few studies directly examining the effect of stavudine dose on treatment outcomes [7,17,24,35,36] particularly from resource-limited settings where stavudine is still widely used [8,37,38]. Our study is the first that we are aware of to examine these effects among a large urban cohort of HIV-infected patients receiving stavudine in this setting.

Compared with a 30 mg dose of stavudine, we found that a 40 mg dose was associated with a high risk of several stavudine-associated adverse events within the first year of treatment. These events included peripheral neuropathy (OR 3.12; 95% CI 1.86-5.25), lipoatrophy (OR 11.8; 95% CI 3.2-43.8) and hyperlactatemia/lactic acidosis (OR 8.37; 95% CI 3.83-18.29). Those receiving the higher dose were also more likely to discontinue stavudine use within the first 12 months on ART (OR 1.71;1 95% CI.13-2.57). Since the 30 mg dose was given later in time, when drugs like tenofovir were more widely available, it is reasonable to be concerned that these results are being driven by the desirability or acceptability of these alternate drugs. However, limiting the analysis to only those who received a non-tenofovir-based regimen gave an adjusted odds ratio of 2.22 (95% CI 1.42-3.48), a stronger effect than the main result for 12-month odds of stavudine substitution, suggesting that this was not the case.

We found no evidence of impaired viral suppression with a 30 mg dose of stavudine. If anything, the estimates suggest the 40 mg dose may be associated with a small increased risk of failure to suppress virus within 12 months (OR 1.62; 95% CI 0.88-2.97) though the imprecise nature of this estimate does not allow us to infer the association with confidence. If, however, a 40 mg dose does actually increase risk of initial failure to suppress virus, then it is possibly due to increased frequency of peripheral neuropathy and other adverse events interfering with adherence [6]. This would mean, moreover, that a 30 mg dose would not sacrifice effectiveness for safety. Rather, it suggests that increasing safety may actually increase effectiveness. In fact, in several of the randomized studies comparing 40 mg and 30 mg stavudine doses, individuals with low body weight received an even further reduced dose of 20 mg of stavudine. Among those with lower body weight, the 20 mg dose could improve safety further without compromising tolerability and effective virologic suppression [35,36].

Newer drugs like tenofovir have become available, and while it appears to be better tolerated than stavudine in terms of several adverse events, dyslipidemias and lipoatrophy in particular [2,8,21], this has been demonstrated largely in comparison to the higher 40 mg dose of stavudine and may not hold true for the lower dose. Data from a randomized trial in Spain comparing a 40 mg stavudine dose with a 30 mg dose or switch to tenofovir demonstrated that both strategies improved peripheral fat and lipid parameters, but only switching to tenofovir was associated with significant reversal of lipoatrophy [16]. Additionally, rates of discontinuation of stavudine (even at the 30 mg dose) are substantially higher than on tenofovir [39].

The 30 mg dose was associated with lower prevalence of lipoatrophy compared with those who had received both 30 mg and 40 mg dosages and individuals receiving AZT in Cameroon [8], although an Asian cohort showed higher discontinuation rates for people taking ZDV versus d4T [40]. A review of nucleoside-switch trials found modest improvement in peripheral wasting after discontinuation of stavudine [41]. Additionally, use of tenofovir can be complicated by toxicity and side effects, including renal insufficiency, osteoporosis and osteopenia. While the risk of renal insufficiency on tenofovir appears to be low and related to pre-existing renal disease [42,43], the limited availability of screening for creatinine clearance and the fact that the cost of this drug is still currently more than five times that of stavudine [44] are important considerations for limiting the use of tenofovir in resource-limited settings.

Our results should be interpreted in light of the potential limitations of the study. First, we studied the effect of stavudine dose only among those with weights of 60 kg or more, and, therefore, these findings may not apply to those with a lower weight at treatment initiation. Second, we performed this analysis on an intent-to-treat basis regarding both weight and dose. While weight and dose may both change after treatment initiation, this was not taken into consideration. However, all outcomes were considered at, or within 12 months of, treatment initiation, and we do not presume to extrapolate these findings beyond this time period.

Third, we were unable to specifically control for the effects of secular trends in care provision and management of adverse events. However, we do note that the largest change in clinical practice in this period was in October 2006 when any fee for clinical care was stopped: while this has a strong effect on loss-to-follow-up rates (data not shown), it had relatively little effect on odds ratios reported here and was controlled for in this analysis, and we believe it unlikely that other secular trends were so confounding as to render these results uninterpretable. Last, the effect of death and loss to follow up were not considered in the primary analysis. While this may have introduced selection bias into the estimates presented, sensitivity analyses showed little difference in estimates when censoring was counted as a negative outcome.

## Conclusions

Our findings from this large cohort offer further evidence that a lower dosage of 30 mg stavudine appears safe and effective within the first year of treatment, and can be implemented in settings where alternate drug options are unavailable. Additional evidence from resource-limited settings is needed to determine whether a routine 20 mg dose of stavudine as part of a HAART regimen is of benefit for patients with low body weights. Lower stavudine dosage is associated with fewer reports of several stavudine-associated adverse events and also a lower risk of stavudine discontinuation within the first year on ART.

## Competing interests

The authors declare that they have no competing interests.

## Authors' contributions

All authors contributed to the conception and design of the study. MM, IS and TM contributed to acquisition of data. DW performed the statistical analysis. MM, MF and DW interpreted the analysis and drafted the manuscript. All authors revised the manuscript critically for intellectual content and have approved the submitted version for publication. All authors read and approved the final manuscript.
